# Early ascertainment of genetic diagnoses clarifies impact on medium-term survival following neonatal congenital heart surgery

**DOI:** 10.1172/JCI180098

**Published:** 2024-07-30

**Authors:** Benjamin J. Landis, Benjamin M. Helm, Matthew D. Durbin, Lindsey R. Helvaty, Jeremy L. Herrmann, Michael Johansen, Gabrielle C. Geddes, Stephanie M. Ware

**Affiliations:** 1 Department of Pediatrics, Riley Hospital for Children,; 2 Department of Medical and Molecular Genetics, and; 3 Department of Surgery, Indiana University School of Medicine, Indianapolis, Indiana, USA.

**Keywords:** Cardiology, Genetics, Cardiovascular disease, Genetic diseases, Surgery

**To the Editor:** While many consider it well established that a genetic diagnosis portends a poor outcome in patients with congenital heart defects (CHDs), prior data have been confounded by highly biased ascertainment and outdated genetic testing methods. The broader clinical genetic testing now available for patients with CHDs has increased early identification of diverse genetic conditions (1). In a time of robust advancement in genetic diagnostics and therapeutics, updated genotype-phenotype understanding is crucial for translating discovery into clinical practice. Currently, the use of genetic testing and geneticist evaluations in infants with CHDs is highly variable, even between cardiac centers with well-developed cardiovascular genetics programs (2). Variations in enrollment ages and follow-up durations further hinder the interpretation of prior outcomes studies (3, 4). Since 2014, our large-volume pediatric cardiac center with statewide catchment has implemented a clinical algorithm to standardize early genetic evaluation of infants with CHD requiring intensive care. This included inpatient consultation by a board-certified medical geneticist upon admission and genetic testing, initially with a chromosomal microarray analysis (CMA) to identify copy-number variants (CNVs) (5). Molecular testing was selectively ordered based on the consulting geneticist’s bedside evaluation and evolved with emergence of broader CHD panels and exome tests. Our systematic clinical initiative provides an opportunity to understand longitudinal survival in critical CHD for a spectrum of genetic diagnoses.

We identified 361 patients who underwent CHD surgery at age 28 days or younger at Riley Hospital from July 1, 2015, to March 31, 2020. Patients were required to have a genetic diagnosis or CMA testing to be included in formal survival analysis (Supplemental Figure 1; supplemental material available online with this article; https://doi.org/10.1172/JCI180098DS1). One patient with trisomy 18 was excluded because of the high mortality rate associated with this diagnosis. We tested the hypothesis that a genetic diagnosis is associated with decreased 1,000-day survival using the univariate log-rank test and multivariate Cox’s proportional hazards regression. A *P* value of less than 0.05 was considered statistically significant.

A genetic diagnosis was identified in 49 of the 299 included patients (16%), consisting of diagnostic CNVs (*n* = 35), aneuploidy (*n* = 9), and single-gene disorders (*n* = 5). The list of genetic diagnoses is provided in Supplemental Table 3. Premature birth was more frequent in patients with a genetic diagnosis compared with patients without a genetic diagnosis (OR 2.18 [95% CI 1.01–4.55], *P* = 0.04). There were no significant differences in sex, race, presence of noncardiac congenital anatomic abnormality (NCAA), use of cardiopulmonary bypass (CPB), or higher STAT mortality risk category operation of 4 or 5 between patients with or without a genetic diagnosis (Supplemental Table 1) (6).

The estimated probability of surviving 1,000 days postoperatively was 0.88 (95% CI 0.85–0.92). Patients with a genetic diagnosis had significantly decreased 1,000-day survival compared with those without a genetic diagnosis (Figure 1). A higher STAT category of 4 or 5 was also significantly associated with decreased survival, while premature birth was not (Supplemental Table 2). Genetic diagnosis (HR 2.3 [1.1–4.7], *P* = 0.025) and STAT category of 4 or 5 (HR 4.1 [1.2–13.3], *P* = 0.02) were independently associated with increased mortality in multivariate Cox regression analysis, which also included year of initial surgery. The proportion of surviving cases with at least 1,000 days of documented follow-up was similar for patients with or without a genetic diagnosis (87% of each group), and follow-up of at least 365 days was above 95% for both groups, indicating good patient retention. Early (30 day) postoperative survival was not statistically different between patients with a genetic diagnosis (96%) and those without a genetic diagnosis (99%) (log-rank *P* = 0.15).

Deaths occurred in 2 of 14 patients with 22q11.2 deletion syndrome, 3 of 6 with trisomy 21, 1 of 3 with CHARGE syndrome, and individual cases of 22q11.2 duplication syndrome, Alagille syndrome, Kabuki syndrome, Williams syndrome, and 9p23-9p13.1 triplication. Recurrent genetic diagnoses without mortality were 15q11.2 (BP1–BP2) deletion syndrome (*n* = 4), 16p11.2 deletion syndrome (*n* = 3), Turner syndrome (*n* = 3), and 8p23.1 duplication syndrome (*n* = 2). Mortality in patients with a CNV of uncertain significance was similar to those with a genetic diagnosis in the early postoperative time period, but patients with a genetic diagnosis continued to experience mortality over longer follow-up (Supplemental Figure 2). Of deaths that occurred more than 100 days after neonatal surgery, an antecedent infection was documented in 5 of 8 cases with a genetic diagnosis versus 1 of 10 without a genetic diagnosis.

This is an observational, single-center analysis focusing on severe CHDs. A minority of patients were excluded for lacking CMA. Nearly half of these excluded patients underwent surgery in 2015, reflecting a short period of time for achieving near-complete adoption of the algorithm. The excluded patients, who underwent operations with risk similar to that of included patients, had relatively low mortality (8%) and less frequent NCAA (Supplemental Table 1). While examination by a medical geneticist, CMA, and selective targeted gene or gene-panel testing based on phenotype was routine, a broader CHD panel or exome sequencing was not routinely performed during the entire study period. The low mortality rate limited the statistical power for detecting differences in 30-day survival between groups, stressing the importance of longitudinal study of well-retained cohorts.

Systematic early genetic evaluation with CMA provides understanding for medium-term survival in critical CHD. Our experience indicates that implementing routine genetic testing in young infants with critical CHD is feasible. Identifying a genetic diagnosis early and understanding specific modifiable risks for early and late complications, such as infection and neurodevelopmental delay, can lead to tailored in-hospital and ambulatory care that optimize longitudinal outcomes. Collaborative initiatives that standardize early genomic testing will advance our developing understanding of the impact of genetic abnormalities in severe CHD.

For further information, see Supplemental Methods.

Values for all data points in graphs are reported in the Supporting Data Values file.

## Supplementary Material

Supplemental data

ICMJE disclosure forms

Supplemental table 3

Supporting data values

## Figures and Tables

**Figure 1:**
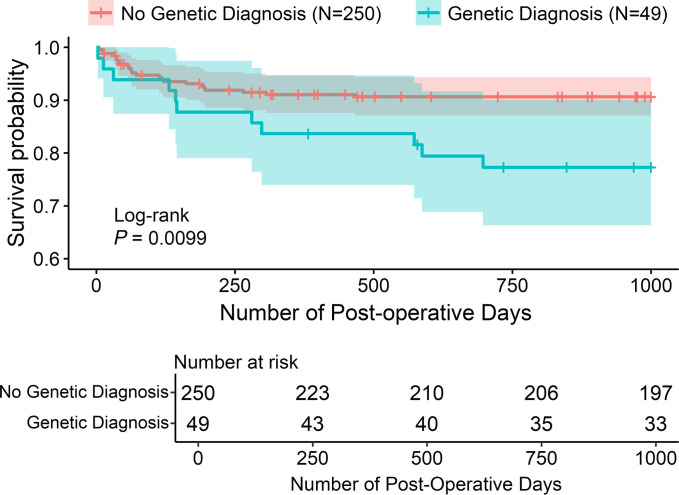
Association between genetic diagnosis and survival in patients undergoing neonatal surgery for CHDs. Kaplan-Meier survival plot stratifying the cohort into genetic diagnosis or no genetic diagnosis groups. Shaded regions indicate 95% CI. *P* value was calculated via log-rank test.

## Version 1 07/30/2024

In-Press Preview

## Version 2 09/17/2024

Electronic publication
