# Supplementary material for: Early ascertainment of genetic diagnoses clarifies impact on medium-term survival following neonatal congenital heart surgery
Source: J Clin Invest. 2024 Jul 30;134(18):e180098. doi: 10.1172/JCI180098 (PMC11405028; doi:10.1172/JCI180098)
Supplement: Supplemental data [file jci-134-180098-s132.pdf]

Supplemental Material

Early ascertainment of genetic diagnoses clarifies impact on medium-term survival following neonatal congenital heart surgery

Benjamin J. Landis[1], Benjamin M. Helm[2], Matthew D. Durbin[1], Lindsey R. Helvaty[2], Jeremy L. Herrmann[3], Michael Johansen[1], Gabrielle C. Geddes[2], Stephanie M. Ware[2]

[1]Department of Pediatrics, Riley Hospital for Children, Indiana University School of Medicine, Indianapolis, IN
[2]Department of Medical and Molecular Genetics, Indiana University School of Medicine, Indianapolis, IN
[3]Department of Surgery, Indiana University School of Medicine, Indianapolis, IN

Corresponding author:
Benjamin J. Landis
1044 W Walnut St, R4-225
Indianapolis, IN 46202
Phone: 317-278-2807
Email: benjland@iu.edu

Contents:
Methods
Supplementary Table 1
Supplementary Table 2
Supplementary Figure 1
Supplementary Figure 2
Author Contributions

Methods

Sex as a biological variable

Our study examined male and female patients, and similar findings are reported for both sexes.

Data collection

Eligible patients were identified by query of our center's cardiothoracic surgery database. Data were collected from STS Society of Thoracic Surgeons (STS) Congenital Heart Surgery Database forms and electronic medical chart review. A patient with trisomy 18 was excluded from survival analysis due to the strong association with decreased survival for this diagnosis. Patients without a genetic diagnosis who did not have CMA testing were excluded from formal analysis in order to ensure consistency in evaluation and minimize the risk of missed diagnoses. Operative risk was defined by the Society of Thoracic Surgeons-European Association for Cardio-Thoracic Surgery (STAT) Mortality Risk Category(5). Vital status and date of last medical encounter were reviewed in December 2022.

Statistics

All analyses were performed using R Statistical Software (v4.3.2). The frequencies of baseline characteristics were compared between groups using Pearson's Chi-squared test. Kaplan-Meier survival analyses including univariate log-rank tests were performed via the *ggsurvfit* R package (v1.0.0) and the *survival* R package (v3.0). Cox proportional hazards regression analyses were performed via the *gtsummary* R package (v1.7.2). P value < 0.05 was considered statistically significant.

Study approval

The study was approved by the institutional review board at Indiana University (Protocol ID: 1408953015) and used a waiver of informed consent. Study procedures were in accordance with institutional guidelines.

Data availability

Data analyzed during this study are included in this published article and values for all data points in graphs are reported in the Supporting Data Values file.

Supplemental Table 1. Comparisons of baseline characteristics between patients with or without a genetic diagnosis (left side of table) and between patients with a genetic diagnosis or negative chromosomal microarray array analysis (CMA) versus patients without CMA testing (right side of table).

Characteristic	Genetic diagnosis (N=49)	Negative CMA (N=250)	Odds ratio [95% confidence interval]	P value		Genetic diagnosis or negative CMA (N=299)	No genetic diagnosis and CMA not sent (N=61)	Odds ratio [95% confidence interval]	P value
Sex male	27 (55%)	146 (58%)	0.87 [0.45-1.71]	0.79		173 (58%)	43 (70%)	0.57 [0.32-1.04]	0.066
Race			NA	0.75				NA	0.84
White	40 (82%)	212 (85%)				252 (84%)	52 (85%)		
Black	7 (14%)	24 (10%)				31 (10%)	7 (11%)		
Asian	1 (2%)	6 (2%)				7 (2%)	2 (3%)		
Native American	0	2 (1%)				2 (1%)	0		
Other	1 (2%)	2 (1%)				33 (1%)	0		
Noncardiac Congenital Anatomic Abnormality*	26 (53%)	97 (39%)	1.78 [0.92-3.47]	0.090		123 (41%)	11 (18%)	3.2 [1.6-6.3]	**0.0007**
Prematurity (GA < 37 weeks)	15 (31%)	42 (17%)	2.18 [1.01-4.55]	**0.040**		57 (19%)	9 (15%)	1.4 [0.6-2.9]	0.43
Cardiopulmonary bypass	17 (35%)	115 (46%)	0.62 [0.31-1.23]	0.19		132 (44%)	31 (51%)	0.8 [0.4-1.3]	0.34
STAT category of 4 or 5	37 (76%)	177 (71%)	1.27 [0.61-2.83]	0.62		214 (72%)	38 (62%)	1.5 [0.9-2.7]	0.15

*Society of Thoracic Surgeons (STS) definition. Statistical comparisons used Pearson's Chi-squared test. Comparisons with P value < 0.05 are in bold. GA: gestational age; NA: not applicable.

Supplemental Table 2. Results of univariate analysis for longitudinal mortality.

Characteristic	P value
**Genetic Diagnosis**	0.0099
No (N=250)	
Yes (N=49)	
**STAT Category**	0.0098
1, 2, or 3 (N=85)	
4 or 5 (N=214)	
**Sex**	0.061
Female (N=126)	
Male (N=173)	
**Prematurity (GA < 37 weeks)**	0.093
No (N=242)	
Yes (N=57)	
**Noncardiac Congenital Anatomic Abnormality***	0.070
No (N=176)	
Yes (N=123)	
**Year of Neonatal Surgery**	0.80
2015 (N=35)	
2016 (N=54)	
2017 (N=52)	
2018 (N=72)	
2019 (N=69)	
2020 (N=17)	

Log-rank tests were used for statistical comparisons between groups.

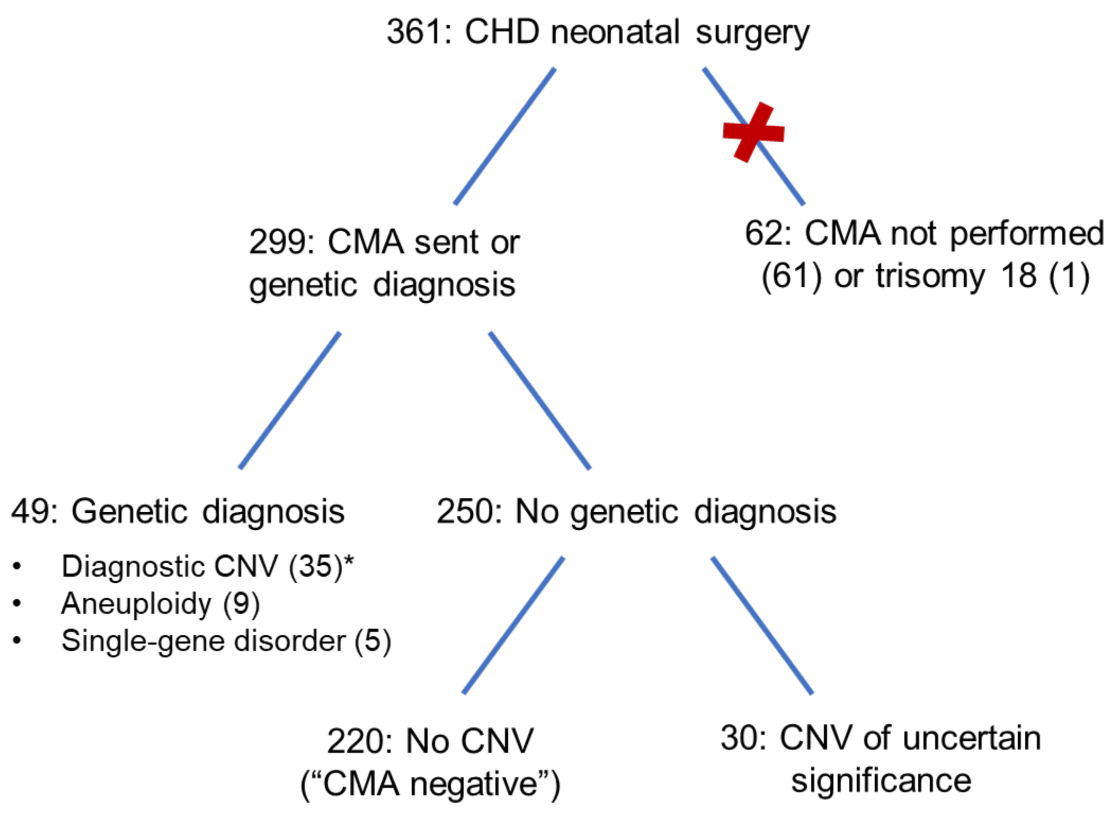

Supplemental Figure 1. Inclusion criteria and genetic categorization of the cohort. Patients who did not have chromosomal microarray analysis (CMA) and one patient with trisomy 18 were excluded from formal survival analysis. The initial congenital heart defect (CHD) operations in the 299 patients included for formal analysis were: Systemic to pulmonary shunt (N=64); Repair of aortic coarctation or interrupted aortic arch (N=56); Pulmonary artery banding (N=51); Norwood procedure (N=35); Arterial switch operation (N=29); Total anomalous pulmonary venous connection repair (N=17); Hybrid stage 1 palliation (N=13); Truncus arteriosus repair (N=7); Other (N=27). *: Includes one patient with Alagille syndrome due to a 7.8 Mb deletion that encompassed the gene *JAG1*.

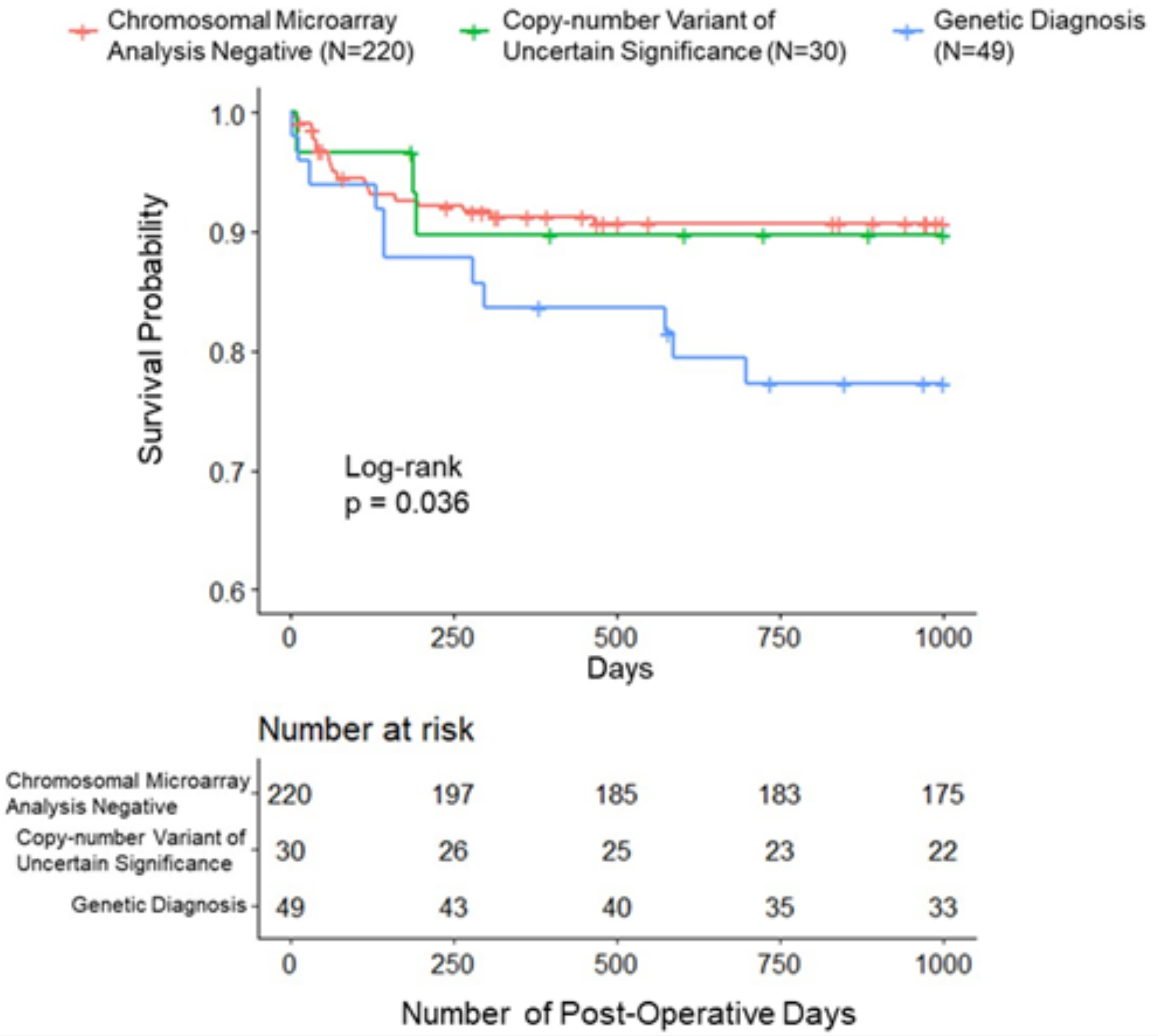

Supplemental Figure 2. Kaplan-Meier survival plot further stratifying the no genetic diagnosis group into patients with negative CMA or copy-number variant of uncertain significance.

Author Contributions:

Designing research study: BJL, BMH, GCG, SMW

Acquiring data: BJL, BMH, MDD, LRH

Analyzing data: BJL

Results interpretation: BJL, BMH, MDD, LRH, JLH, MJ, GCG, SMW

Writing and/or critically revising manuscript: BJL, BMH, MDD, LRH, JLH, MJ, GCG, SMW
